# Effect of Growth Hormone Treatment on Growth Rates in Patients With Growth Hormone Deficiency, Idiopathic Short Stature, and Small for Gestational Age

**DOI:** 10.7759/cureus.73571

**Published:** 2024-11-13

**Authors:** Lale Guliyeva, Ismail Dundar, Aysehan Akinci, Harika Gozde Gozukara Bag

**Affiliations:** 1 Department of Pediatric Endocrinology, İnönü University, Malatya, TUR; 2 Department of Biostatistics and Medical Informatics, İnönü University, Malatya, TUR

**Keywords:** childhood, growth hormone deficiency, growth hormone therapy, height sds, height velocity

## Abstract

Objective: This study aimed to determine the response to growth hormone (GH) therapy of patients with growth hormone deficiency (GHD), idiopathic short stature (ISS), and small for gestational age (SGA).

Materials and methods: The data of the 202 children who received GH treatment were analyzed retrospectively. Laboratory parameters, puberty stages, annual growth rates, body mass index (BMI), GH side effects, target height (TH), estimated adult height (EAH), and bone age (BA) were obtained during the GH treatment period.

Results: Out of 202 cases, 121 were girls (59.9%) and 81 were boys. Among these cases, 69 (34.1%) had partial growth hormone deficiency (PGHD), 43 (21.2%) had complete growth hormone deficiency (CGHD), 37 (18.3%) had ISS, and 53 (26.2%) were born SGA. The average age at the onset of treatment was 11.2±2.67 years. The height standard deviation score (SDS), height velocity, and predicted adult height (PAH) of the patients with PGHD, CGHD, and SGA values were significantly higher than ISS patients at the end of the third year of a similar dose of GH treatment. The highest growth velocity was determined in the group with CGHD, while the lowest height velocity was found in patients with ISS during three years of the treatment.

Conclusion: It was determined that GH treatment is an efficient and reliable therapy method for improving short stature in patients with PGHD, CGHD, ISS, and SGA. We observed a higher growth rate in patients with PGHD, CGHD, and SGA than in the ISS group with standard dose GH treatment.

## Introduction

Short stature can be defined as a child's height below -2 standard deviations (SD) of the value previously determined in the population for sex and age. The child's height below -3 SD according to the specified criteria indicates severe short stature [1,2]. The diagnosis of growth hormone deficiency (GHD) is based on an inadequate growth hormone (GH) response to at least two pharmacological GH stimulation tests (clonidine, levodopa, insulin stimulation, etc.). Several pathological conditions affecting the GH-insulin-like growth factor-1 axis (GH-IGF-1 axis) can lead to GH deficiencies. It may be isolated GHD or combined with other pituitary hormone deficiencies. The degree of deficiency may be partial or complete, or the physiological release of GH may be impaired. Physiological secretion disorder of GH or biological inactive GH pathologies should be considered in growth retardations with a positive response to GH stimulation tests and minimum IGF-1 levels [1,3].

Short stature that develops without a known underlying cause such as a systemic, chromosomal, endocrine, or nutritional problem is defined as ISS. Recombinant growth hormone (r-GH) therapy is used in GHD, synthesis, secretion, or dysfunction disorders. Clinical data showed that short children born SGA who fail in their catch-up growth by two to four years of age and children with ISS benefit from r-GH therapy even if they have no GHD [1,2,4]. Our purpose in this study is to determine the response to GH treatment of patients with growth retardation according to the diagnostics distribution during the three-year treatment period who are followed in our outpatient clinic.

## Materials and methods

A total of 202 patients were diagnosed with partial growth hormone deficiency (PGHD), complete growth hormone deficiency (CGHD), idiopathic short stature (ISS), and small for gestational age (SGA), who were followed up regularly in our outpatient clinic because of short stature. Age at admission, history and family history, gestational week, birth weight, anthropometric measurement values, maternal and paternal heights, target heights, target height standard deviation score (SDS), physical examination findings, puberty stages, laboratory, and the results of two separate GH stimulation tests performed at different time intervals and radiological imaging findings were recorded. The height of the subjects was measured with a wall-mounted stadiometer (Seca® brand; Seca GmbH & Co. KG, Hamburg, Germany) while standing in a neutral position, with heels together, and no shoes. The body weight of the patients was measured with a digital scale (Fakir Hausgerate brand) sensitive to 100g-150kg, while the patients were wearing thin clothes and without shoes, by placing them on the scale with both feet on the center of the scale. Body mass index (BMI) was calculated using this formula: body weight (kg)/height (m²). The evaluation of height and BMI SDS was calculated according to the data of Turkish children in the study of Neyzi et al. [5]. To determine the target height prediction, for girls: [((father's height-13 cm) + mother's height)/2], for boys: [((mother's height + 13) + father's height)/2] formulas were used. Bone age (BA) was determined by a pediatric endocrinologist using the Greulich & Pyle atlas from the left hand-wrist radiographs. In cases with BA greater than six years of age, the predicted adult height (PAH) was calculated according to BA using the Bayley-Pinneau method [6]. The staging of pubertal development was based on breast development in girls and testicular volume in boys according to Tanner and Marshall criteria [7,8]. Both testicular sizes were measured and recorded separately with the "Prader orchidometer" [9]. Patients with chronic diseases affecting growth, Turner syndrome, Noonan syndrome, Prader-Willi syndrome, SHOX gene mutation, and patients with skeletal dysplasia were excluded from the study.

Serum hormone levels were measured with a chemiluminescent immunoassay (immune chemiluminescent microparticle assay, or ICMA) on a Siemens 2000 immunoassay analyzer (Beckman Coulter Inc., CA, USA). Anthropometric measurements are compatible with GHD: (a) Height below 3 percentile according to chronological age (according to Neyzi standards); (b) Growth rate according to chronological age -2.5 SDS or less; (c) BA is two years or more retarded than chronological age. GH stimulation tests (clonidine, levodopa) were applied as a screening test to patients with growth retardation and low serum basal IGF-1 and insulin-like growth factor-binding protein 3 (IGFBP-3) levels [10]. GH treatment was initiated when GH levels were found below 10 ng/mL in two GH stimulation tests performed on all patients. GH response to pharmacological stimuli was evaluated: (a) Maximum stimulated GH response ≥10 ng/mL: normal, (b) Maximum stimulated GH response 5.1-10 ng/mL: PGHD, (c) Maximum stimulated GH response ≤ 5 ng/mL: CGHD. Patients with normal birth weight, normal body proportions, normal GH response to stimulation tests, height SDS corresponding to mean parental SDS, and no identified cause for their short stature were defined as ISS. Short children born SGA and fail in their catch-up growth by two to four years of age were included in the study as SGA group. According to the diagnoses of the patients, they were divided into groups such as PGHD, CGHD, ISS, and SGA. Anthropometric measurements (height SDS, Δ height SDS, height velocity (growth velocity between years), Δ (height SDS-target height SDS), PAH), laboratory results were recorded at the time of diagnosis and the first, second, and third years of treatment.

Statistical analysis

Data from 202 children who received GH treatment between January 2002 and February 2019 were retrospectively analyzed. The data was analyzed by IBM SPSS Statistics for Windows version 22.0 (IBM Corp., Armonk, NY, USA) statistical program. The measured data were shown with maximum, median, and minimum values. Kruskal-Wallis analysis of variance was used to compare grouped data and the Conover test for pairwise comparison of groups. In the same group, the Friedman test between different years and the Conover test was used to compare the years. The alpha (significance level) was accepted as 0.05 in the tests.

Ethical approval

The study proposal was approved by the Ethical Committee of İnönü University Faculty of Medicine (approval number: 2018/21-14).

## Results

Of 202 cases, 81 were male and 121 (59.9%) were female. The age range of the cases was 1-16 years and the mean age at presentation was 7.88±3.55 years. PGHD was found in 69 (34.2%) cases, CGHD in 43 (21.3%), SGA in 53 (26.2%) and ISS in 37 (18.3%) cases. In the birth history of the cases, 26 (12.9%) were premature (<37 weeks), 172 (85.1%) were mature (37-42 weeks), and two (1.0%) were post-term (>42 weeks). Of these patients, 53 (26.2%) were SGA and 144 (71.2%) were normal birth weight. At the time of diagnosis, 165 cases (81.7%) were prepubertal and 37 cases (18.3%) were pubertal. GH was applied at an instead of 31.03 mcg/kg/day. There was no statistically significant difference between the groups in terms of GH doses at the time of diagnosis and the first, second, and third years of treatment.

**Figure 1:**
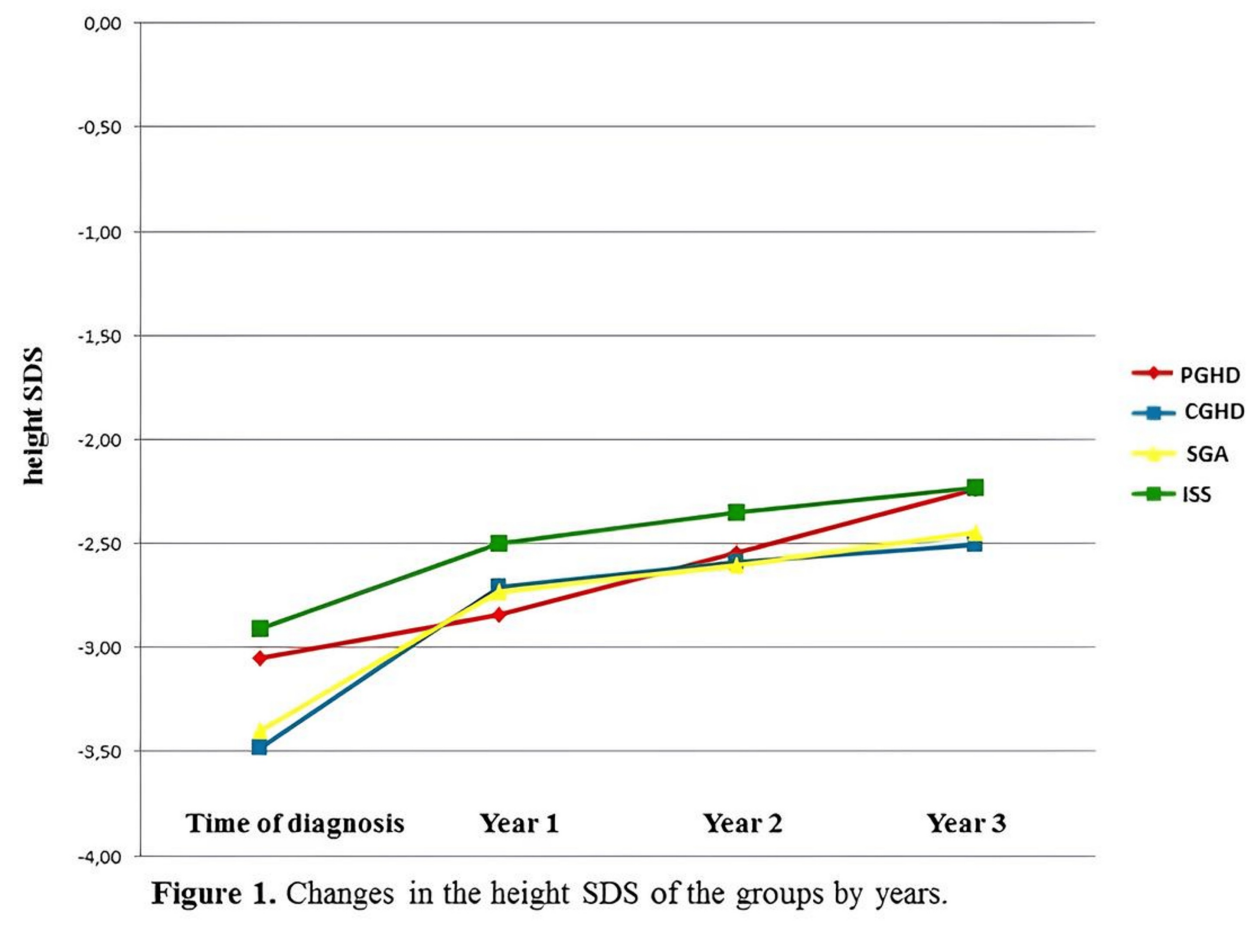
Changes in the height SDS of the groups by years SDS: standard deviation score; PGHD: partial growth hormone deficiency; CGHD: complete growth hormone deficiency; SGA: small for gestational age; ISS: idiopathic short stature

A significant increase in the height SDS was found in the PGHD, CGHD, and SGA groups during the three-year treatment period (Table 1). Height SDS changes of the groups according to the years are shown in Figure 1 and Δ height SDS within the groups and in their annual follow-up are shown in Table 2. In patients with CGHD and SGA, Δ height SDS values ​​in the first year of treatment were found to be significantly higher in prepubertal cases than in pubertal cases (p=0.048).

**Table 1:** Δ height SDS values of the cases during the treatment according to the diagnoses NS: not significant; Δ: difference; SDS: standard deviation score; PGHD: partial growth hormone deficiency; CGHD: complete growth hormone deficiency; SGA: small for gestational age; ISS: idiopathic short stature p^a^: Comparison of height SDS values between the time of diagnosis and the first year of treatment p^b^: Comparison of height SDS values between the time of diagnosis and the second year of treatment p^c^: Comparison of height SDS values between the time of diagnosis and the third year of treatment

Diagnosis	Time of diagnosis	p-value	Year 1	p-value	Year 2	p-value	Year 3
	Mean		Mean		Mean		Mean
PGHD	-3.05 (-3.68 to -2.60)	NS	-2.84 (-3.70 to -1.60)	NS	-2.54 (-3.3 to -1.48)	p^c^=0.04	-2.24 (-3.04 to 1.35)
CGHD	-3.48 (-6.15 to -1.07)	NS	-2.71 (-4.23 to -0.54)	p^b^=0.02	-2.59 (-3.75 to -0.47)	p^c^=0.02	-2.51 (-3.8 to -0.17)
SGA	-3.40 (-5.10 to -1.82)	p^a^=0.01	-2.73 (-4.69 to -0.25)	p^b^=0.01	-2.60 (-5.2 to -0.60)	p^c^=0.01	-2.45 (-5.27 to -1.08)
İSS	-2.91 (-3.53 to -2.31)	NS	-2.50 (-3.39 to -2.14)	NS	-2.35 (-3.38 to -1.91)	NS	-2.23 (-3.5 to -1.28)

**Table 2:** Δ height SDS values of the cases during the treatment according to the diagnoses NS: not significant; SDS: standard deviation score; Δ: difference; PGHD: partial growth hormone deficiency; CGHD: complete growth hormone deficiency; SGA: small for gestational age; ISS: idiopathic short stature p^1^: Comparison of Δ height SDS values between PGHD and CGHD at the first year of treatment p^a^: Comparison of Δ height SDS values between the first year and third year of treatment

Diagnosis	Year 1	Year 2	p-value	Year 3
	Mean	Mean		Mean
PGHD	0.18 (-0.97 to 1.10)	0.10 (-0.1 to 1.28)	NS	0.21 (-1.81 to 0.83)
CGHD	0.76 (-0.55 to 2.67)	0.27 (-1.80 to 2.12)	NS	0.29 (-2.37 to 0.88)
SGA	0.47 (-0.8 to 1.57)	0.12 (-0.83 to 1.70)	p^a^=0.007	0.10 (-0.80 to 2.42)
ISS	0.34 (-2.98 to 2.08)	0.22 (-1.26 to 1.10)	NS	0.23 (-0.48 to 0.81)
p-value	p^1^=0.007	NS		NS

**Figure 2:**
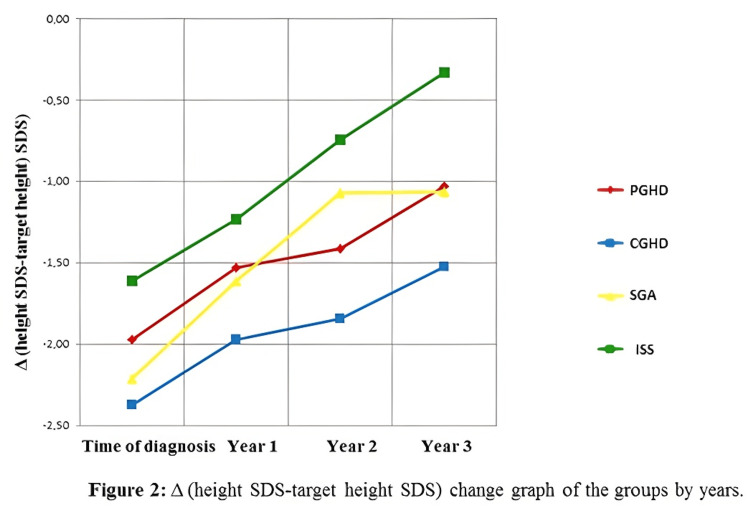
Graph showing the change in Δ (height SDS-target height SDS) across groups over the years SDS: standard deviation score; Δ: difference; PGHD: partial growth hormone deficiency; CGHD: complete growth hormone deficiency; SGA: small for gestational age; ISS: idiopathic short stature

**Figure 3:**
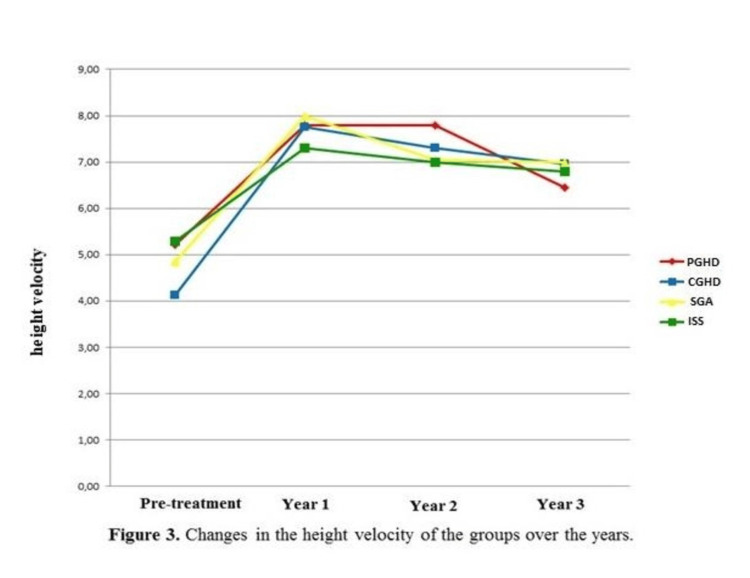
Changes in the height velocity of the groups over the years PGHD: partial growth hormone deficiency; CGHD: complete growth hormone deficiency; SGA: small for gestational age; ISS: idiopathic short stature

At the end of the third year, the Δ height (height SDS-target height SDS) value of the groups increased according to the beginning of the treatment, they benefited from the treatment without any significant change in GH doses over the three years. We also observed that all groups approached the target height SDS, more clearly in the ISS group, during three years of treatment (Table 3). Among the prepubertal and pubertal subgroups of patients with PGHD and ISS, the Δ (height SDS-target height SDS) value at the first year of treatment was found to be lower in the prepubertal group. The change in Δ height (height SDS-target height SDS) of the groups by years is shown in Figure 2. When the annual height velocity within each group was analyzed, although it was determined that the height velocity increased in the third year in all groups according to the time of diagnosis, a statistically significant increase was found only in the CGHD group (Table 4). In patients with PGHD, the annual height velocity in the third year of treatment was found to be significantly higher in prepubertal cases than in pubertal cases (p=0.038). Annual height velocities are shown in Figure 3. At the end of the third year, it was determined that the PAH values ​​of the groups increased in all groups according to the time of diagnosis (Table 5).

**Table 3:** Δ height SDS values of the cases during the treatment according to the diagnoses NS: not significant; SDS: standard deviation score; Δ: difference; PGHD: partial growth hormone deficiency; CGHD: complete growth hormone deficiency; SGA: small for gestational age; ISS: idiopathic short stature p^2^: Comparison of Δ (height SDS-target height SDS) values between CGHD and ISS groups at the second year of treatment p^3^: Comparison of Δ (height SDS-target height SDS) values between CGHD and ISS groups at the third year of treatment p^a^: Comparison of Δ (height SDS-target height SDS) values between the time of diagnosis and the second year of treatment p^b^: Comparison of Δ (height SDS-target height SDS) values between the time of diagnosis and the third year of treatment

Diagnosis	Time of diagnosis	Year 1	p-value	Year 2	p-value	Year 3
	Mean	Mean		Mean		Mean
PGHD	-1.97 (-3.28 to -0.71)	-1.53 (-3.27 to -0.29)	p^a^=0.03	-1.41 (-1.75 to -0.15)	p^b^=0.03	-1.03 (-2.36 to -0.17)
CGHD	-2.37 (-6.0 to -0.74)	-1.97 (-4.35 to -0.12)	NS	-1.84 (-3.8 to -0.17)	p^b^=0.001	-1.52 (-3.15 to -0.05)
SGA	-2.21 (-4.10 to -0.33)	-1.61 (-3.93 to 0.37)	p^a^=0.003	-1.07 (-3.99 to 0.62)	p^b^=0.001	-1.06 (-3.90 to 0.97)
İSS	-1.61 (-2.64 to -0.22)	-1.23 (-2.30 to -0.33)	NS	-0.74 (-2.70 to 0.01)	p^b^=0.017	-0.33 (-2.82 to 0.81)
p-value	NS	NS		p^2^=0.007		p^3^=0.04

**Table 4:** Δ (height SDS-target height SDS) of the cases during the treatment according to the diagnoses NS: not significant; Δ: difference; PGHD: partial growth hormone deficiency; CGHD: complete growth hormone deficiency; SGA: small for gestational age; ISS: idiopathic short stature p^a^: Comparison of height velocity values between pre-treatment and first year of treatment p^b^: Comparison of height velocity values between pre-treatment and second year of treatment p^c^: Comparison of height velocity values between pre-treatment and third year of treatment

Diagnosis	Height velocity pre-treatment	p-value	Year 1	p-value	Year 2	p-value	Year 3
	Mean		Mean		Mean		Mean
PGHD	5.21 (2.70-6.50)	p^a^=0.02	7.80 (6.00-12.50)	p^b^=0.02	7.80 (6.10-10.00)	NS	6.45 (1.20-8.90)
CGHD	4.13 (2.00-5.40)	p^a^=0.02	7.75 (4.40-10.00)	p^b^=0.01	7.30 (4.10-12.00)	p^c^=0.01	6.95 (4.50-9.00)
SGA	4.86 (2,00-6,50)	p^a^=0.01	8.00 (6.40-11.30)	NS	7.05 (2.00-9.40)	NS	7.00 (3.50-10.00)
ISS	5.28 (3.60-7.20)	NS	7.30 (0.80-10.70)	NS	7.00 (1.00-9.30)	NS	6.80 (4.00-9.30)

**Table 5:** PAH values of the cases during the treatment according to the diagnoses NS: not significant; PAH: predicted adult height; PGHD: partial growth hormone deficiency; CGHD: complete growth hormone deficiency; SGA: small for gestational age; ISS: idiopathic short stature p^a^: Comparison of PAH values between the time of diagnosis and the second year of treatment p^b^: Comparison of PAH values between the time of diagnosis and the third year of treatment p^c^: Comparison of PAH values between first and second year of treatment p^d^: Comparison of PAH values between first and third year of treatment

Diagnosis	Time of diagnosis	Year 1	p-value	Year 2	p-value	Year 3
	Mean	Mean		Mean		Mean
PGHD	149.7 (131.0-173.6)	153.0 (132.7-168.9)	p^a^=0.001, p^c^=0.008	156.7 (134.0-175.0)	p^b^=0.001, p^d^=0.043	157.1 (134.2-173.6)
CGHD	147.5 (134.0-168.5)	151.5 (138.0-169.0)	p^a^=0.021	154.3 (141.0-172.0)	p^b^=0.001	155.5 (138.0-174.0)
SGA	146.0 (140.0-163.0)	151.0 (141.4-172.0)	NS	152.0 (143.3-170.0)	p^b^=0.003	153.0 (143.4-168.0)
İSS	152.0 (139.3-170.0)	153.0 (143.0-172.0)	NS	155.0 (144.7-175.0)	p^b^=0.001, p^d^=0.016	158.5 (147.9-181.0)

Although it was determined that IGF-1 values ​​increased in all groups in the third year according to the time of diagnosis, a significant increase was found in the CGHD and ISS groups (Table 6). Among the prepubertal and pubertal subgroups of patients with SGA, IGF-1 SDS values ​​at the time of diagnosis were found to be lower in prepubertal cases than in pubertal cases. There was no statistically significant difference between the groups in IGFBP-3 SDS values ​​at the time of diagnosis and the first, second, and third years of treatment. In the cranial MR examinations performed during the follow-up of the patients in our study group, the most common cranial pathology was 8.4% (n= 17) pituitary microadenoma and the second rank was partial empty sella with 5.9% (n=12). Partial empty sella was more common in patients with CGHD (14.6%, n=6). In our study, side effects such as enlarged nevi, pain in the knees, enlarged clavicle, enlarged nose, enlarged hands, and feet, tympanic membrane perforation, pituitary adenoma enlargement, and increased cancer antigen (CA) markers were observed in patients who received GH treatment. No life-threatening or other serious side effects were observed.

**Table 6:** Change of IGF-1 SDS values of patient groups during treatment NS: not significant; IGF-1: insulin-like growth factor 1; SDS: standard deviation score; PGHD: partial growth hormone deficiency; CGHD: complete growth hormone deficiency; SGA: small for gestational age; ISS: idiopathic short stature p^1^: Comparison of IGF-1 SDS values between CGHD and SGA groups at the time of diagnosis p^a^: Comparison of IGF-1 SDS values between the time of diagnosis and the second year of treatment p^b^: Comparison of IGF-1 SDS values between the time of diagnosis and the third year of treatment

Diagnosis	Time of diagnosis	Year 1	p-value	Year 2	p-value	Year 3
	Mean	Mean		Mean		Mean
PGHD	-0.94 (-2.50 to 3.89)	0.33 (-2.96 to 5.33)	NS	0.64 (-1,57 to 6.50)	NS	1.17 (-0.70 to 2.14)
CGHD	-1.67 (-2.92 to 0.63)	-0.58 (-3.20 to 8.80)	p^a^=0,001	0.18 (-0.91 to 7.31)	p^b^=0.008	0,63 (-1.95 to 4.26)
SGA	-0.89 (-3.60 to 4.47)	-0.80 (-3.00 to 3.34)	NS	-0.23 (-2.90 to 6.5)	NS	-0.14 (-2.80 to 2.91)
ISS	-1.38 (-3.01 to -0.40)	-0.23 (-2.00 to 0.84)	NS	-0.22 (-1.40 to 1.53)	p^b^=0.029	0.60 (-3.60 to 3.36 )
p-value	p^1^=0.021	NS		NS		NS

## Discussion

The distribution of diagnoses among the cases was as follows: 69 (34.2%) PGHD, 43 (21.3%) CGHD, 37 (18.3%) ISS, and 53 (26.2%) SGA were detected. In terms of gender distribution, 144 cases (63.7%) were girls and 82 (36.3%) were boys. The average age at presentation was 7.88±3.55 years, 176 (87%) were prepubertal and 26 (13%) were pubertal at the time of diagnosis. The majority of our study group consisted of PGHD. In a study from Italy, the mean age at diagnosis was 9.6, most of them were prepubertal, and 59% were male. Of these patients, 85.5% GHD, 3.0% SGA, 0.3% ISS, and the rest received GH treatment for other reasons [11]. In a similar study from Spain, the average age at presentation was 9.8 years, mostly prepubertal and 56% male. A total of 78.1% of these cases were followed with GHD, 10.9% SGA, 0.9% ISS, and the remaining patients were followed up with other diagnoses [12]. In Canada, most of them were prepubertal, the age at presentation was 8.5 years, and 50.7% were male. It has been reported that 61.9% of these cases received GH treatment, 2.2% SGA, 4.5% ISS and the rest received GH treatment for other reasons [13]. Most of the prepubertal patients who received GH treatment in Korea were found to be 8.49 years old and 51.8% male at the time of presentation. Of the patients, 64% were followed up with GHD, 15.5% ISS, 10.1% SGA, and the remaining patients with other diagnoses [14]. In our study, female patients were in the majority. Consistent with the literature, most of our cases were patients with a diagnosis of GHD, but our cases with a diagnosis of ISS were at a higher rate compared to the studies mentioned above. It has been reported that the earlier the treatment is started, the better the response to treatment [15,16]. In our study, it was observed that the time of initiation of GH treatment in patients was earlier than in other studies. This can be explained by the easier access to health systems in our country and the fact that the reimbursement of drugs is made by the Ministry of Health.

Studies have shown that GH treatment increases growth rate in the short term, has a dose-response relationship in the first two years of treatment, has a positive effect on final height in the long term, and has good reliability over a wide dose range [17]. In GHD cases treated with GH, the most important factor affecting the final height is the height gained with treatment in the prepubertal period. It has been reported that the highest height SDS gain was found in the CGHD group, and the lowest gain was found in the ISS group [13,18-22]. In a study from the USA, patients with isolated GHD among patients who received GH therapy responded better than those with ISS. In this study, although a higher dose of GH was used in patients with ISS than in other groups, it was observed that these patients benefited the least from GH treatment [21]. In a study from Korea, it was reported that the GHD group benefited more from the treatment among the different patients receiving GH treatment [14]. In a study conducted in Japan, the mean height SDS value of the complete GHD group receiving GH treatment increased more than the PGHD group [22].

Low-dose GH was administered to our patients at an average of 0.22 mg/kg/week (31 mcg/kg/day). There was no significant difference between the GH treatment doses between the groups and in the same group by years. In some studies in the literature, higher doses of GH were used in cases diagnosed with SGA and ISS, while in other studies, similar doses of GH were used in all groups, similar to our study [13,14,23]. In our study, Δ height SDSs and growth rate in patients with PGHD, CGHD, and SGA were significantly increased during three-year treatment (Figures 1-3 and Tables 1-4). Among the groups, the highest height SDS gain was found in the CGHD group, and the lowest gain was found in the ISS group. These results can be explained by the mild GH resistance described in some ISS patients and the fact that although high doses of GH are recommended for this group, they were treated with standard doses of GH in our study. We observed that our patients with CGHD give the best response to GH treatment consistent with the other studies in the literature. In many studies, it has been reported that the CA/BA ratio is higher in patients with GHD compared to ISS patients and decreases with GH treatment and an increase in PAH is achieved [14,24-27]. In our study, when the annual follow-up of each group was examined, at the end of the third year, it was determined that the CA/BA ratio decreased in the CGHD, SGA, and ISS groups. It was determined that BA approached the chronological age with GH treatment. Consistent with the literature, a significant increase in PAH values was found in the follow-up with GH treatment in patients with PGHD, CGHD, SGA, and ISS. In these patients, it was determined that the increase in PAH value with GH treatment developed in parallel with the increase in growth rate and height SDS value, and the decrease in the CA/BA ratio.

In the literature, while a decrease in BMI SDS value was detected during GH treatment in patients with GHD, an increase in this value was reported in patients without GHD [21,28]. In our study, the BMI SDS value in all patients was found to be within the normal range before treatment and during follow-up, but it was determined that the BMI SDS value decreased significantly in the PGHD and CGHD groups, there was no significant difference between the two groups. This was consistent with other studies in the literature that found a decrease in BMI SDS values ​​with GH treatment in patients with GHD. No significant difference was found in BMI SDS values ​​between prepubertal and pubertal groups in all groups at the follow-up and the end of treatment in our patients.

It has been reported that monitoring of serum IGF-1 levels during GH treatment is particularly significant in terms of patient compliance with treatment and keeping the treatment within safe limits. In the GeNeSIS study from Japan, it was reported that the mean IGF-1 SDS and IGFBP-3 SDS values of patients diagnosed with GHD increased at the end of the fourth year of treatment [22]. In our study, it was determined that the IGF-1 SDS values between the groups were lower in the CGHD group than in the SGA group at the time of diagnosis. There was no significant difference between the groups in the first, second, and third years of the treatment. When the annual follow-ups of each group were examined, although a tendency to improve in IGF-1 SDS values was detected in all groups, the increase in only CGHD and ISS groups was statistically significant (Table 6).

Side effects observed during the use of recombinant GH have been reported in many studies. In a study conducted in Italy, side effects such as headache, hypothyroidism, hypogonadism, secondary hypothyroidism, hypoglycemia, diarrhea, and urticaria were reported in patients treated with GH [11]. In a study from Spain, 93 of the 1143 patients treated with GH experienced undesirable side effects. Seven patients with serious side effects were identified, two of which were associated with GH [12]. In our study, the side effects were observed as enlargement of the nevi in two patients, two patients with knee pain, one patient with clavicle enlargement, two patients with an enlarged nose, three patients with hands and feet growth, one patient with eardrum perforation, two patients with enlargement of pituitary adenoma, and one patient with increased CA marker who received GH treatment. In our study, the side effects detected during the treatment were evaluated as mild and few in accordance with the literature.

In recent studies, it has been reported that MRI plays an important role in the diagnosis of GHD. Various studies have reported that some structural disorders such as pituitary stem agenesis, anterior pituitary hypoplasia, and posterior pituitary ectopia are detected in MRI [29-32]. In the KİGS (Pfizer International Growth Database) study, various abnormal MRI findings were detected in 4032 (26.8%) of 15043 children who underwent MRI research. Primarily, abnormalities detected in MRI are pituitary hypoplasia and HME (hypoplastic anterior pituitary, missing pituitary stalk, and ectopic posterior pituitary) [29]. The frequency of detecting pathologies in the pituitary gland has been reported to vary between 12% and 86% in other studies. Such a wide range may be due to differences in the interpretation of pituitary MRIs and the use of contrast agents [33-36]. In our study, cranial pathology was detected in 22 (19.6%) patients diagnosed with PGHD and CGHD. While the most common cranial pathology in the literature was pituitary hypoplasia, the most common pathology in our study was partial empty sella. This was interpreted to mean that the MRI findings of both pathologies may have been evaluated differently because of the similarity.

## Conclusions

In conclusion, GH treatment is an efficient and safe treatment method for improving short stature in patients with PGHD, CGHD, ISS, and SGA. We observed that a better growth rate was achieved with standard dose GH treatment in PGHD, CGHD, and SGA groups compared to ISS.
